# Microglial phenotypes and toll-like receptor 2 in the substantia nigra and hippocampus of incidental Lewy body disease cases and Parkinson’s disease patients

**DOI:** 10.1186/s40478-014-0090-1

**Published:** 2014-08-07

**Authors:** Karlijn J Doorn, Tim Moors, Benjamin Drukarch, Wilma DJ van de Berg, Paul J Lucassen, Anne-Marie van Dam

**Affiliations:** Swammerdam Institute for Life Sciences, Center for Neuroscience, University of Amsterdam, Science Park 904, 1098 XH Amsterdam, The Netherlands; Department Anatomy and Neurosciences, VU University Medical Center, Neuroscience Campus Amsterdam, Van der Boechorststraat 7, 1081 BT Amsterdam, The Netherlands

**Keywords:** Parkinson’s disease, Substantia nigra, Hippocampus, Incidental Lewy body disease, Microglia, Toll-like receptor 2

## Abstract

Next to α-synuclein deposition, microglial activation is a prominent pathological feature in the substantia nigra (SN) of Parkinson’s disease (PD) patients. Little is known, however, about the different phenotypes of microglia and how they change during disease progression, in the SN or in another brain region, like the hippocampus (HC), which is implicated in dementia and depression, important non-motor symptoms in PD.

We studied phenotypes and activation of microglia in the SN and HC of established PD patients (Braak PD stage 4–6), matched controls (Braak PD stage 0) and of incidental Lewy Body disease (iLBD) cases (Braak PD stage 1–3) that are considered a prodromal state of PD. As recent experimental studies suggested that toll-like receptor 2 (TLR2) mediates α-synuclein triggered microglial activation, we also studied whether TLR2 expression is indeed related to pathology in iLBD and PD patients.

A clear α-synuclein pathology-related increase in amoeboid microglia was present in the HC and SN in PD. Also, morphologically primed/reactive microglial cells, and a profound increase in microglial TLR2 expression were apparent in iLBD, but not PD, cases, indicative of an early activational response to PD pathology. Moreover, TLR2 was differentially expressed between the SN and HC, consistent with a region-specific pattern of microglial activation.

In conclusion, the regional changes in microglial phenotype and TLR2 expression in primed/reactive microglia in the SN and HC of iLBD cases indicate that TLR2 may play a prominent role in the microglial-mediated responses that could be important for PD progression.

## Introduction

Parkinson’s disease (PD) is a progressive neurodegenerative disorder that affects 1–2% of the elderly population [1]. Besides classical motor problems that are related to nigro-striatal dopamine deficits [2], also non-motor symptoms are common in PD. These include autonomic dysfunction, sensory, sleep and cognitive disturbances as well as neuropsychiatric alterations, that strongly affect the quality of life of PD patients [3–8]. An important pathological hallmark of PD is the presence of α-synuclein inclusion bodies, i.e. Lewy bodies (LBs) and Lewy neurites (LNs), which spread in a predictable manner throughout the brain [9]. While their spatiotemporal development parallels the appearance of non-motor and motor symptoms [10], little is known about changes in microglial activation, another prominent pathological feature of PD [11–13].

Under neurodegenerative conditions, microglial cells readily transform from a ramified morphology into amoeboid-shaped cells. They acquire specific functions, including phagocytosis, and can secrete a variety of factors, such as cytokines, chemokines, reactive oxygen species (ROS) and trophic factors [14,15]. While *in vitro* and experimental animal studies had demonstrated that α-synuclein can trigger microglial activation, *in vivo* imaging and post-mortem immunohistochemical studies have now established the presence of amoeboid microglia and pro-inflammatory mediators in the substantia nigra (SN) in PD [11,16–20]. Interestingly, increased densities of amoeboid microglia were also observed in the olfactory bulb (OB) of PD patients and in a mouse model for PD (1-methyl-4-phenyl-1,2,3,6-tetrahydropyridine) [21,22,23,24]. Thus, microglial activation occurs also outside the SN, where it coincides with α-synuclein deposition. In contrast to the SN, it is not associated with neuronal cell death [23]. These observations agree with novel views on microglia as a heterogeneous cell population that may exert brain region-dependent functions [13,25,26,27]. As such, microglia are thought to contribute to local inflammatory responses, not in a uniform, but rather in a brain region-specific manner.

Based on their morphology and receptor expression, different microglial phenotypes have been identified in the brain [28,29]. These include cells with a small cell body and many thin ramifications, called ramified microglia, cells with a larger, less round cell body and thick ramifications, called primed and reactive microglia, and cells with hardly or no ramifications, which are classified as amoeboid microglia [29]. Besides these different morphologies, that likely reflect different functionalities [30–32], also toll-like receptors (TLR) have attracted considerable attention. TLR activation of microglia forms the primary response against a wide array of pathogens [33,34], including aggregated proteins, e.g. α-synuclein [34,35]. Activation of the TLR pathway increases the expression of various pro-inflammatory cytokines, like IL-6, IL-1 and TNF-α [36,37].While earlier evidence has been obtained in the field of Alzheimer’s disease (AD) [38,39] and Multiple Sclerosis (MS) [40], recent studies have implicated TLRs also in the pathogenesis of PD [41–44]. In support of this, TLR-deficient mice are less vulnerable to MPTP toxicity and show decreased microglial activation in the SN after MPTP treatment [45], while (Thy1)-[A30P] α-synuclein transgenic mice show a significant upregulation of TLR2 [46]. Moreover, the oligomeric form of α-synuclein acts as an endogenous agonist of TLR2 on microglia where it stimulates pro-inflammatory cytokine expression [47]. As this effect is not induced by TLR3 and TLR4, the TLR2 subtype is considered highly specific for the neuroinflammatory response of microglia in PD [41].

Thus far, studies on microglia in PD have focused mainly on the SN. Based on the spatiotemporal development of α-synuclein pathology over different brain regions during PD progression, also microglial activation may differ between brain regions. In the present study, we investigated differences in microglial phenotype, i.e. in ramified, primed/reactive and amoeboid subtypes, and compared the SN to the hippocampus (HC), a brain region that has been implicated in the cognitive deficits and depressive symptoms frequently present in PD [48,49]. We studied tissue of clinically diagnosed and neuropathologically verified PD patients (Braak PD stage 4–6) [50], age- and gender-matched control subjects (Braak PD stage 0) and incidental Lewy body disease (iLBD) cases (Braak PD 1–3), that did not have clinical PD symptoms but displayed α-synuclein deposition at autopsy and can therefore be considered a prodromal state of PD [51]. Moreover, as α-synuclein can stimulate microglia through TLR2, we further investigated whether TLR2 is expressed in the SN and HC of iLBD and PD cases in relation to α-synuclein deposition.

## Materials & methods

### Post-mortem brain tissue

Human post-mortem brain tissue was obtained from the Netherlands Brain Bank (NBB, Amsterdam, The Netherlands) or from the department of Pathology, VU University Medical Center (VUmc, Amsterdam, The Netherlands). In compliance with all local ethical and legal guidelines, informed consent for brain autopsy and the use of brain tissue and clinical information for scientific research was given by either the donor or the next of kin. The SN and HC were included from 14 clinically diagnosed and neuropathologically verified PD patients (Braak PD stage 4–6). Of these, 8 SN and 9 HC were included from patients who had suffered from dementia during PD progression (PDD). Furthermore, from iLBD cases (Braak PD stage 1–3), 9 SN and 6 HC were studied. The control group consisted of healthy subjects without neurological or psychiatric disease and without LB pathology (Braak PD stage 0), of which 13 SN and 9 HC were studied. Sections from all donors were included in the studies performed. Importantly, in 75% of the cases, the HC and SN tissue was obtained from the same patient, ruling out the possibility that regional differences are due to ‘between patient’ differences. All three groups were matched for gender and age; control subjects ranged from 62–92, iLBD cases from 56–91 and PD patients ranged from 59–96 years of age. Furthermore, all subjects were controlled and matched for post-mortem delay and cerebrospinal fluid pH value. Donors who died of sepsis, or stroke were excluded (Table 1).

**Table 1:** **Clinical and neuropathological information of all included subjects**

				**Braak staging**				
**C#**	**Sex**	**Age**	**PMD (hrs)**	**AD (NFT / Aβ)**	**PD (α-syn)**	**D**	**Region**	**Cause of death**
1	F	92	7:00	1A	0	NDC	HC + SN	Acute death, pulmonary emboly
2	M	88	4:23	2A	0	NDC	HC + SN	Gastro-intestinal bleeding
3	F	84	6:55	1O	0	NDC	HC + SN	Myelodysplasia
4	M	82	5:10	1O	0	NDC	HC + SN	Unknown
5	M	62	7:20	1O	0	NDC	HC + SN	Unknown
6	F	77	2:55	1B	0	NDC	SN	Pulmonary metastasis
7	M	84	5:35	1A	0	NDC	SN	Heart failure
8	M	75	4:15	1B	0	NDC	SN	Respiratory insufficiency
9	M	71	6:00	2(−)	0	NDC	SN	Respiratory insufficiency
10	M	81	7:55	2O	0	NDC	SN	Renal insufficiency
11	F	78	7:30	2(−)	0	NDC	SN	Heart failure
12	M	79	6:00	1B	0	NDC	SN	Metastates prostate and lung cancer
13	F	83	8:00	1O	0	NDC	SN	Respitatory insuffisiency
14	F	83	3:20	1B	0	NDC	HC	Legal Euthanasia
15	F	84	4:45	1O	0	NDC	HC	Heart failure
16	F	85	5:19	2B	0	NDC	HC	Natural death, pulmonary disease
17	M	78	<17:00	1O	0	NDC	HC	Natural cause,
18	F	82	5:10	2O	2	iLBD	HC + SN	Heartfailure
19	M	86	4:00	2B	1	iLBD	HC + SN	Respiratory insufficiency
20	M	56	5:00	0(−)	2	iLBD	HC + SN	Pneunomia
21	F	91	4:50	(−)	1	iLBD	HC + SN	Exhaustion, colon carcinoma
22	M	84	7:20	1B	3	iLBD	HC + SN	Prostate cancer
23	F	82	7:00	1O	3	iLBD	SN	Pneunomia
24	F	85	4:40	2A	1	iLBD	SN	Dehydration
25	F	93	<10:00	0(−)	1	iLBD	SN	Unknown
26	F	78	<10:00	0(−)	1	iLBD	SN	Unknown
27	M	87	10:20	1A	1	iLBD	HC	Pneunomia, heart failure
28	M	83	4:50	1A	4	PDD	HC + SN	Heartfailure
29	F	59	9:35	1A	4	PD	HC + SN	Shock due to blood loss in digestive tract
30	F	90	4:50	1B	4	PDD	HC + SN	Unknown
31	M	84	9:00	1A	5	PDD	HC + SN	Pneunomia and dehydration
32	F	70	7:05	2B	6	PDD	HC + SN	Haematemesis by oesophagitis
33	F	87	5:25	2B	6	PDD	HC + SN	Pneunomia
34	M	73	5:35	1A	5	PDD	HC + SN	Direct cause unknown (morphine)
35	M	83	5:15	1B	6	PDD	HC + SN	Pneunomia
36	F	84	7:25	2B	5	PD	HC + SN	Old age, shortness of breath
37	F	96	7:10	1B	5	PD	HC + SN	Old age
38	M	86	5:10	2B	5	PD	HC + SN	Heartfailure
39	M	71	5:50	1A	6	PD	HC + SN	Respiratory failure
40	M	86	5:35	1O	4	PD	SN	Aspiration pneumonia
41	M	80	7:05	1B	6	PDD	SN	Unknown, possibly urine tract infection
42	F	83	6:05	1O	4	PDD	HC	Cachexia by dementia, infarction
43	M	83	6:35	1B	6	PDD	HC	Pneunomia

D, clinical diagnosis; PMD, post mortem delay; NFT, neurofibrillary tangles; Aβ amyloid beta; α-syn, alpha synucleine; NDC, non-demented control subject; iLBD, incidental Lewy Body Disease cases; PD, Parkinson’s disease; PDD, Parkinson’s disease with dementia; HC, hippocampus, SN, substantia nigra.

The Braak neurofibrillary tangles (NFT) stages and Aβ scores for AD, and LBs/LNs containing α-synuclein score for PD were provided by the NBB and were based upon careful neuropathological evaluation of disease-relevant brain areas by established and qualified neuropathologists [52]. The density and distribution of LBs/LNs, NFT and Aβ plaques were determined based on Bodian silver staining and immunohistochemical analysis of α-synuclein (Clone KM51, Novacastra, Bioconnect BV), hyperphosphorylated tau (Clone AT8, Pierce, Rockford, IL) and Aβ (Clone 6 F/3D, DAKO, DakoCytomation BV), respectively. The AD Braak stages and scores were further matched between control subjects, iLBD cases and PD patients, ruling out any possible differences in microglial activation due to AD pathology. The clinicopathological data of the patients including the Braak staging for PD and AD of all donors is summarized in Table 1.

### Tissue processing

At autopsy, brain regions were dissected and immersion-fixed in 4% formaldehyde for four weeks and subsequently embedded in paraffin. From the paraffin blocks of the HC and the ventral mesencephalon, which included the SN pars compacta, 10 micrometer (μm) thick sections were cut with a microtome, mounted on positively-charged glass slides (Menzel-Glaser SuperFrost Plus, Braunschweig, Germany) and dried in a stove overnight at 37°C before immunohistochemical staining.

### Immunohistochemistry

Sections were heated in a stove for one hour at 56°C, before they were deparaffinized in xylene and rehydrated through a graded series of ethanol (100%, 96%, 90% and 70%, respectively) and distilled water. For subsequent antigen retrieval, sections were rinsed in 10 mM Tris Buffer (pH 9.0) containing 1 mM EDTA (Tris-EDTA) and placed in preheated Tris-EDTA buffer in a steamer at 90-99°C for 30 minutes. For α-synuclein staining, antigen retrieval was performed using pretreatment with 98% formic acid (Sigma, Steinheim, Germany) for 10 min at room temperature (RT). After pretreatment, the sections were RT, rinsed in Tris-buffered saline (TBS, pH 7.6) and incubated for 20 min in TBS containing 0.3% H_2_O_2_ and 0.1% sodiumazide to block endogenous peroxidase activity. Non-specific binding was blocked with 5% non-fat dried milk in TBS containing 0.5% Triton (TBS-T, pH 7.6; blocking solution) for 30 min at RT. Subsequently, sections were incubated overnight at 4°C with mouse anti-CD68, mouse anti-α-synuclein, goat anti-TLR2 or rabbit anti-Ionized calcium binding adaptor molecule 1 (Iba1) antibodies diluted in blocking solution (details on the primary antibodies are specified in Table 2). Sections were then washed in TBS and incubated for 2 hr at RT in the appropriate dilutions of biotinylated goat anti mouse IgG’s, goat anti rabbit IgG’s or donkey anti goat IgG’s (1:400; Jackson ImmunoResearch Laboratories Inc., West Grove, Pennsylvania, USA) followed by horse radish peroxidase (HRP)-labeled avidin-biotin complex (ABC complex, 1:400; Vector Laboratories, Burlingame, CA, USA) in TBS-T for 1 hr at RT. Iba1 and CD68 staining were visualized using 3,3-diaminobenzidine (DAB, Sigma, St.Louis, USA) and counterstained with heamatoxylin, while TLR2 and α-synuclein immunostaining were visualized using DAB-nickel as a chromogen, and counterstaining was performed with Fast red. After dehydration in graded ethanol solutions, sections were cleared in xylene and coverslipped in Entellan (Merck).

**Table 2:** **Primary antibodies used for single labeling**

**Antigen**	**Species**	**Final dilution**	**Source**
Human CD68	Mouse	1:500	DAKO, clone KP1
Human α-synuclein	Mouse	1:2000	BD-Bioscience, 610786
Human Iba1	Rabbit	1:4000	WAKO chemicals
Human TLR2	Goat	1:2000	R&D systems

### Immunofluorescence

For double-immunofluorescent labeling of glial cells and TLR2 expression, sections were co-incubated with combinations of antibodies against microglia, i.e. CD68, Iba1, or astrocytes, i.e. GFAP, and against TLR2. Antigen retrieval was performed by pretreating the sections with Tris-EDTA (pH 9.0) and all antibodies (TLR2/Iba1, TLR2/CD68, TLR2/GFAP) were diluted in blocking solution, as indicated above. After an overnight incubation at 4°C, the sections were washed and subsequently incubated for 2 h at RT with the appropriate Alexa Fluor 488 or Alexa Fluor labeled 594 IgG’s (1:400, Jackson Immunoresearch, Westgrove, PA, USA) (see Table 3 for information on antibodies and conjugates). After washing, sections were coverslipped with Vectashield and later examined using a confocal microscope (Leica TSC-SP2-AOBS; Leica Microsystems, Wetzlar, Germany).

**Table 3:** **Antibodies and conjugates used for double labeling**

**Antigen**	**Species**	**Final dilution**	**Source**	**Secondary ab’s and conjugates**
Human Iba1	Rabbit	1:2000	WAKO chemicals	DoaR-AF488 1:400
Cow GFAP	Rabbit	1:2000	DAKO	DoaR-AF488 1:400
Human CD68	Mouse	1:300	DAKO, clone KP1	DoaM-AF488 1:400
Human TLR2	Goat	1:500	R&D systems	DoaG-AF594 1:400

### Microglial phenotypes: identification criteria for ramified, primed/reactive and amoeboid microglia

Classification of ramified and amoeboid microglia morphology was performed as described in detail before [23]. Microglial cells were identified by positive CD68 immunoreactivity (IR) [53]. Both microglial subtypes are characterized by cytoplasmic staining, but the ramified microglial celltype can be distinguished by its small cell body and thin, radially projecting processes. Amoeboid microglia are characterized by a densely CD68-stained cell body typically surrounded by no or only very few short/stump processes [23]. Furthermore, we defined Iba1 positive ramified microglia as cells having a small circular body with highly ramified processes. Iba1 positive microglia with a primed/reactive phenotype display a bigger and less round cell body with thicker and sometimes less ramifications compared to the ramified phenotype. Iba1 positive amoeboid microglia show an amoeboid cell body with, at most, two unramified processes, or they are completely devoid of them [29].

### Semi-quantitative analyses of microglial cells, a-synuclein deposition and TLR2 expression

For semi-quantificative analysis, the numbers of CD68 positive amoeboid microglial cells and α-synuclein immunopositive deposits (LBs and LNs) present in the HC pyramidal cell layers, i.e. CA4, CA3, CA2 and CA1, were counted per CA area (region of interest, ROI, 0.5 mm^2^) at a 10 × 3.3 magnification. For standardization purposes, hippocampal sections were collected around the anterior-to-midlevel of the HC of every subject, and only when large DG and CA subregions were both present. For the SN, the area of interest was larger (ROI, 1.5 mm^2^) and quantification was performed at the anatomical levels of the oculomotor nerve. Microglial cells were counted at a 10 × 3.3 magnification; hence, these data are presented as number of cells per 1.5 mm^2^. Semi-quantitative analysis of TLR2 and Iba1 IR was based on the amount of IR per ROI. To this end, pictures were taken at a 10×3.3 magnification and on these a standardized threshold procedure was used that distinguished background from specific IR. Subsequently, the amount of specific IR was measured within the defined ROI, and expressed as percentages of TLR2 and Iba1 IR per ROI. Semi-quantitative analyses were performed unbiased using Cell^F^ Olympus Soft Imaging Solutions GmbH software, version 3.1 (Tokyo, Japan). All data were expressed as mean +/− standard error of the mean (SEM).

### Statistical analysis

All statistical analyses were performed with the SPPS package version 20.0 (Statistical Product and Service Solutions, Chicago, IL, USA). The normal distribution of the data was investigated using the Kolmogorov-Smirnov test. When normally distributed (Iba1), statistical analysis for between group effects was performed with an independent sample T-test. When a normal distribution was absent (CD68, TLR2, α-synuclein), statistical analyses were executed with the non-parametric Kruskal-Wallis test to examine main group effects between control subjects, iLBD cases and PD patients. Subsequently, Mann–Whitney U tests were performed as post-hoc tests to assess between group effects. Statistical analyses for the different subregions of the HC within the same cases were performed with the non-parametric paired Wilcoxon test. Significance was set at 0.05 with Bonferroni corrections for multiple testing where applicable.

## Results

### α-Synuclein pathology and increased numbers of CD68 positive amoeboid microglia in the SN

**Figure 1:**
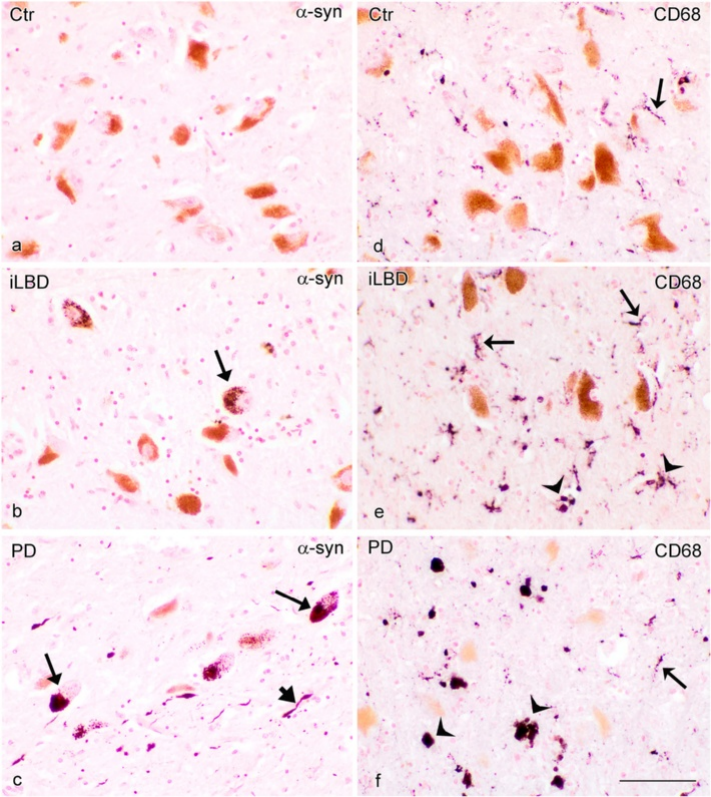
**α-Synuclein pathology and CD68 immunopositive microglia in the substantia nigra (SN) of control subjects, iLBD cases and PD patients.** Brown staining depicts the melanin-containing, dopaminergic neurons that degenerate during PD. **(a)** No intraneuronal α-synuclein immunoreactivity (IR)(purple) was observed in the SN of control subjects, **(b)** some intraneuronal α-synuclein IR was found in the SN of iLBD cases (arrow), and **(c)** widespread intraneuronal and neuritic α-synuclein IR is observed in the SN of PD patients (LBs: arrows, LNs arrowhead); **(d)** a control subject showing some CD68 positive ramified microglial cells (purple, arrow), **(e)** an iLBD case showing prominent CD68 positive ramified (arrow) and amoeboid (concave arrowhead) microglial phenotypes, and **(f)** a PD patient, showing few CD68 positive ramified (arrow), but many more amoeboid microglial phenotypes (concave arrowhead); bar **(a-f)** = 100 μm.

**Figure 2:**
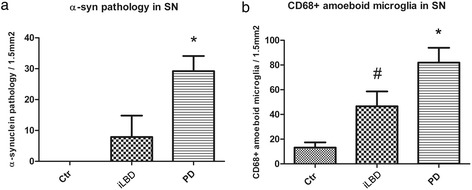
**Quantification of α-synuclein pathology and CD68 immunopositive amoeboid microglia density in the substantia nigra (SN) of control subjects, iLBD cases and PD patients. (a)** A significant increase was present in α-synuclein deposits in PD patients compared to the control and iLBD cases (*p < 0.001 vs Ctr, iLBD), and **(b)** a significant increase in CD68 positive amoeboid microglia was present in PD patients compared to control subjects and iLBD cases (*p < 0.01 vs Ctr; *p < 0.05 vs iLBD). The number of CD68 positive amoeboid microglia in iLBD cases was also significantly higher compared to control subjects (^#^p < 0.01 vs Ctr). Data represent mean ± SEM.

We first examined the SN that is classically affected in PD. In the SN of control subjects and iLBD cases, numerous neuromelanin-containing and pigmented dopaminergic neurons were present (Figure 1). No α-synuclein pathology was observed in the SN of controls, while in the iLBD cases, few α-synuclein positive deposits were detected (Figure 1a, b respectively; Figure 2a). In contrast, PD patients showed extensive loss of neuromelanin-containing, dopaminergic neurons in the SN, and the number of LBs and LNs was significantly increased compared to both control and iLBD cases (Figure 1c; Figure 2a; PD vs Ctr/iLBD p < 0.01; Ctr n = 13, Mean = 0 ± 0; iLBD n = 9, Mean = 7 ± 6.2; PD n = 14, Mean = 29.2 ± 4.9). Furthermore, in control subjects, the large majority of CD68 positive microglia had long and fine processes, indicative of their ramified phenotype, with only a small number of cells displaying an amoeboid morphology (Figure 1d). The number of amoeboid microglia was significantly increased in iLBD cases compared to control subjects (Figure 1e; Figure 2b; iLBD vs Ctr p = 0.002; Ctr Mean = 13.2 ± 4.1; iLBD Mean = 46.6 ± 12.0). PD patients showed numerous and widespread CD68 positive amoeboid microglia in the SN, significantly more than in the iLBD and in control cases (Figure 1f; Figure 2b; PD vs iLBD p = 0.035, PD vs Ctr p = 0.000; PD Mean = 80.6 ± 12.9).

### α-Synuclein pathology is apparent in the hippocampal CA2 subregion of PD patients

**Figure 3:**
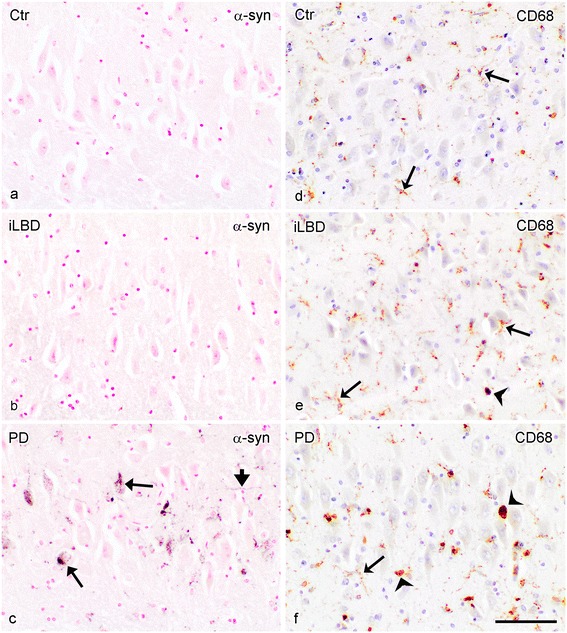
**α-Synuclein pathology and CD68 immunopositive microglia in the hippocampal CA2 region of control subjects, iLBD cases and PD patients. (a, b)** α-synuclein IR in the CA2 of control and iLBD cases is absent compared to **(c)** PD patients (purple, LBs: arrow, LNs: arrowhead); **(d)** a control subject showing CD68 positive ramified microglial cells (arrow), **(e)** an iLBD case showing CD68 positive ramified (arrow) and few microglial cells with an amoeboid (concave arrowhead) phenotype and **(f)** a PD patient, showing CD68 positive ramified (arrow) and several amoeboid (concave arrowhead) microglial cells; bar **(a-f)** = 100 μm.

**Figure 4:**
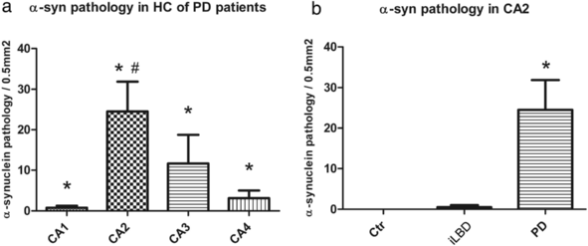
**Quantification of α-synuclein pathology in the hippocampus (HC) of control subjects, iLBD cases and PD patients. (a)** Significant differences were present in α-synuclein deposits between all pyramidal subregions of the HC in the PD patients (*p < 0.05). The CA2 region was affected the most relative to the other hippocampal subregions (^#^p < 0.01). **(b)** Comparison of α-synuclein pathology in CA2 between the three patient cohorts showed a significant increase in the PD patients compared to control and iLBD cases (*p < 0.01 vs iLBD, Ctr). Data represent mean ± SEM.

Next, α-synuclein pathology was examined in the HC. No α-synuclein IR was present in the HC of control and iLBD cases (shown for CA2; Figure 3a, b). In contrast, α-synuclein positive deposits were prominent in the HC of PD and PDD patients and especially concentrated in the pyramidal layer CA2, where their numbers were significantly increased compared to the CA2 of control and iLBD cases (Figure 3c; Figure 4b; PD vs Ctr/iLBD p < 0.01; Ctr n = 9, Mean 0 ± 0; iLBD n = 6, Mean 0.5 ± 0.5; PD n = 14 Mean 24.5 ± 7.4) and also compared to the other pyramidal regions CA1, CA3 and CA4 within PD patients (Figure 3c; Figure 4a; significantly different between all regions p < 0.05; and CA2 vs CA1/CA3/CA4 p = 0.001; CA2 Mean = 24.5 ± 7.4; CA1 Mean = 0.8 ± 0.5; CA3 Mean = 4.9 ± 2.5; CA4 Mean = 3.1 ± 1.9).

### Increased number of CD68 positive amoeboid microglia in the HC of iLBD cases and PD patients

**Figure 5:**
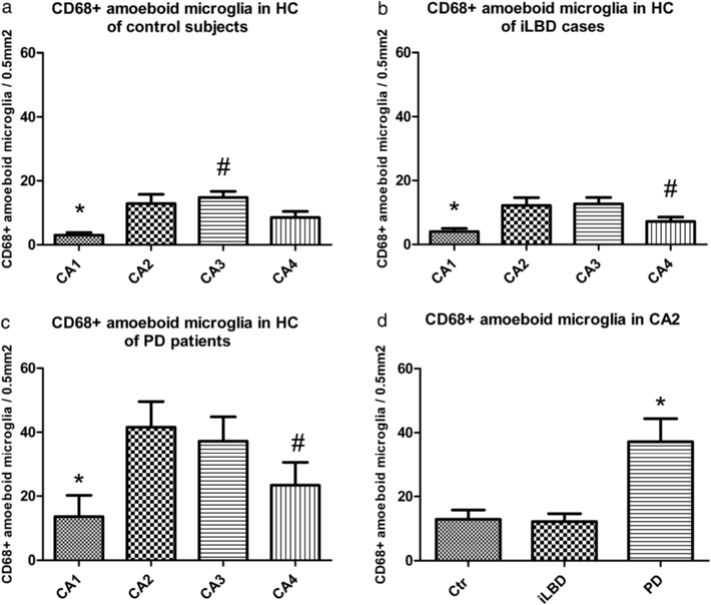
**Quantification of CD68 immunopositive amoeboid microglia in the hippocampus (HC) of control subjects, iLBD cases and PD patients. (a-c)** Numbers of CD68 positive amoeboid microglia were significantly lower in CA1 compared to all other pyramidal layers in **(a)** the control subjects, **(b)** iLBD cases and **(c)** PD patients (*p ≤ 0.02 vs CA2, CA3, CA4). A significant increase was observed in **(a)** CA3 compared to CA4 of control subjects (^#^p < 0.05 vs CA4), and in **(b, c)** CA2 and CA3 compared to CA4 in **(b)** iLBD cases and **(c)** PD patients (^#^p < 0.05, ^#^p ≤ 0.01, respectively vs CA2, CA3). **(d)** Comparison of all groups together resulted in a significant increase in the numbers of CD68 positive amoeboid microglia in the PD patients relative to control and iLBD cases (data shown for CA2, *p < 0.01 vs iLBD, Ctr). Data represent mean ± SEM.

In the pyramidal layer of the HC of control subjects, iLBD cases and PD patients, CD68 positive, ramified microglia were present (shown for CA2 Figure 3d-f), but CD68 positive amoeboid microglia were clearly more present in PD patients (Figure 3f) compared to control and iLBD cases (Figure 3d, e), especially in the CA2 and CA3 region (shown for CA2 Figure 5d; PD vs Ctr/iLBD p = 0.007; Ctr Mean = 13 ± 2.9; iLBD Mean = 12.1 ± 2.5; PD Mean = 37.2 ± 7.2). Interestingly, in control and iLBD cases, a significantly higher number of CD68 positive amoeboid microglia was present in the CA2 and CA3 as compared to other subregions (Figure 5a, b; Ctr: CA1 vs CA2/CA3/CA4 p < 0.02, CA3 vs CA4 p = 0.025; CA1 Mean = 2.7 ± 0.8; CA2 Mean = 12.9 ± 2.9; CA3 Mean = 14.8 ± 1.9; CA4 Mean = 8.5 ± 1.9; iLBD: CA1 vs CA2/CA3/CA4 p = 0.027, CA4 vs CA2/CA3 p < 0.05; CA1 Mean = 4.0 ± 1.0; CA2 Mean = 12.2 ± 2.5; CA3 Mean = 12.7 ± 2.0; CA4 Mean = 7.2 ± 1.4). This subregion-specific difference in CD68 positive amoeboid microglia was larger in PD patients, with the highest numbers present in the CA2 region (Figure 5c; PD: CA1 vs CA2/CA3/CA4 p < 0.01, CA4 vs CA2/CA3 p < 0.01, CA3 vs CA2 p = 0.278 n.s.; CA1 Mean = 7.0 ± 1.2; CA2 Mean = 37.2 ± 7.2; CA3 Mean = 32.5 ± 6.5; CA4 Mean = 17.5 ± 4.5). Based on the α-synuclein and CD68 observations in the HC, our subsequent analyses of Iba1 and TLR2 immunoreactivity focused mainly on the CA2 region.

### Iba1 positive microglia in SN and HC in PD

**Figure 6:**
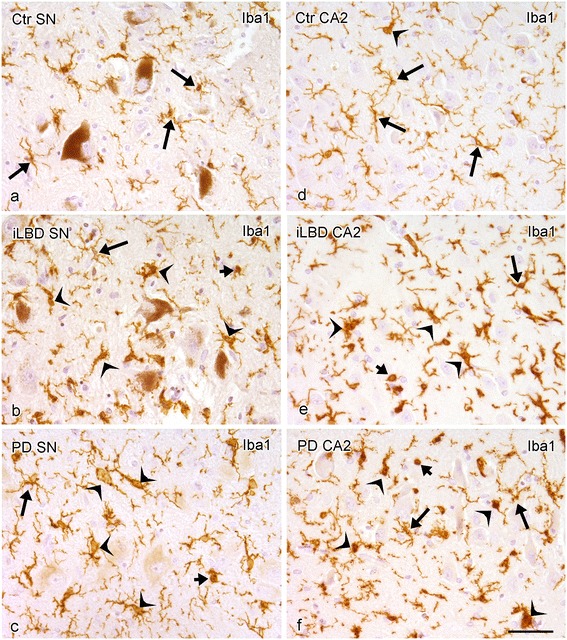
**Iba1 immunopositive microglia in the substantia nigra (SN) and hippocampal CA2 region of control subjects, iLBD cases and PD patients. (a,b)** Widely distributed Iba1 positive ramified microglial cells (arrow) in a control subject, **(c,d)** an iLBD case and **(e,f)** a PD patient. Iba1 positive microglia are present with ramified (arrow), primed/reactive (concave arrowhead) and amoeboid (short arrow) phenotypes; bar **(a-f)** = 50 μm.

In addition to CD68, Iba1 IR was used to determine the overall presence of microglial cells and to classify different phenotypes in the SN and HC. In all subjects, Iba1 positive ramified microglia displayed a small circular cell body with highly ramified processes. This phenotype was the most prominent one in control subjects (Figure 6a, d). In iLBD cases, additional primed/reactive Iba1 positive microglial cells were present, with a bigger and less round cell body and thicker ramifications as compared to the ramified phenotype (Figure 6b, e). These primed/reactive microglia were also present in PD patients with the addition of a few Iba positive amoeboid microglia displaying at most two unramified processes, or none at all (Figure 6c, f).

**Figure 7:**
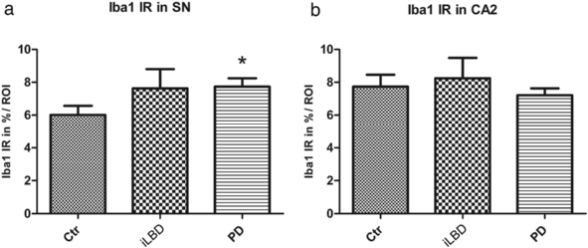
**Quantification of Iba1 immunoreactivity (IR) in the substantia nigra (SN) and hippocampal CA2 region of control subjects, iLBD cases and PD patients. (a)** A significant increase was present in Iba1 IR in the SN of PD patients relative to control subjects (*p < 0.05 vs Ctr); **(b)** no significant difference was present in the CA2. Data represent mean ± SEM.

A significant increase was found in total Iba1 density in the SN of PD patients relative to controls (Figure 6a, c; Figure 7a; PD vs Ctr p = 0.03; Ctr Mean = 6.0 ± 0.6; PD Mean = 7.7 ± 0.5), but not in iLBD cases compared to controls. Total Iba1 density did not differ between subregions of the HC (not shown) nor between control subject, iLBD cases and PD patients (shown for CA2 Figure 6d-f; Figure 7b; Ctr Mean = 7.7 ± 0.7; iLBD Mean = 8.3 ± 1.2; PD Mean = 7.2 ± 0.4).

### Increased TLR2 expression in the SN and HC of iLBD cases

**Figure 8:**
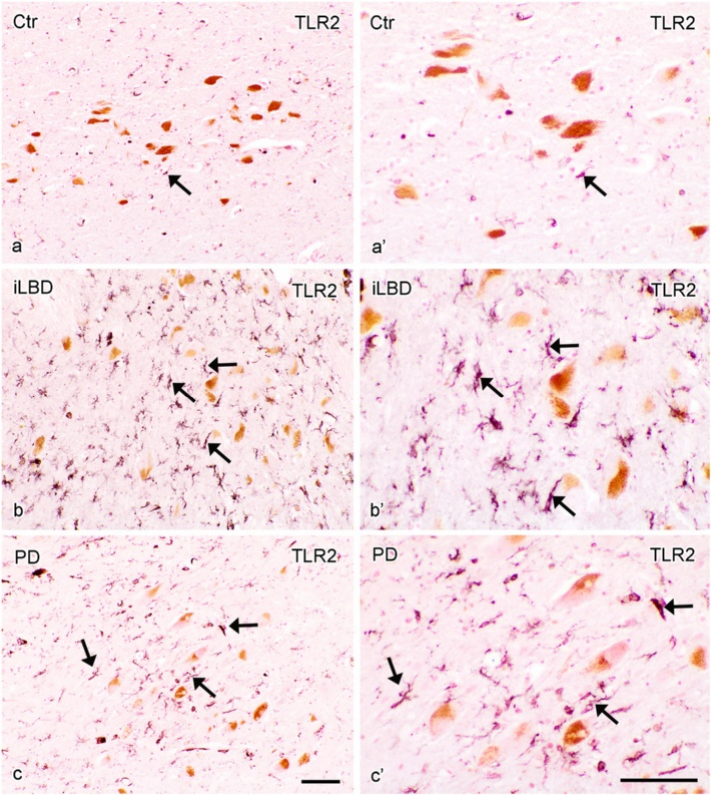
**TLR2 immunopositive cells in the substantia nigra (SN) of control subjects, iLBD cases and PD patients. (a)** Control subject showing few TLR2 positive cells (arrow), **(b)** an iLBD case showing widespread and numerous TLR2 positive cells (arrows) and **(c)** a PD patient, showing moderate numbers of TLR2 positive cells (arrows), **(a’-c’)** represent higher magnifications of **(a-c)**; bar **(a-c, a’-c’)** = 100 μm.

**Figure 9:**
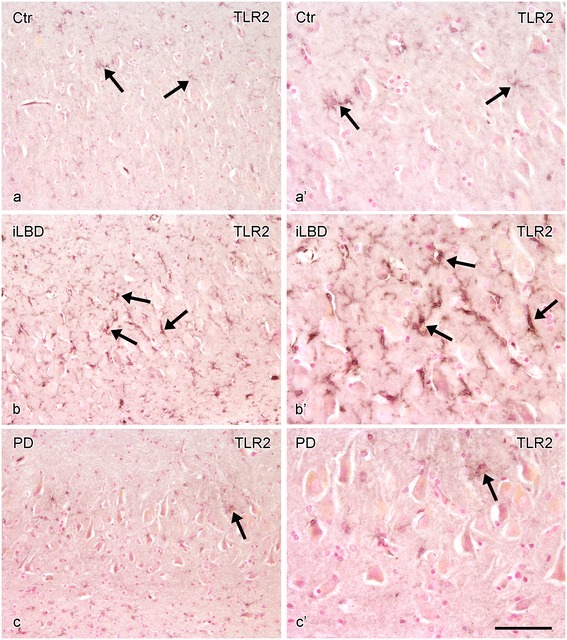
**TLR2 immunopositive cells in the hippocampal CA2 region of control subjects, iLBD cases and PD patients. (a)** A control subject showing few TLR2 positive cells (arrow), **(b)** an iLBD case showing widespread and numerous TLR2 positive cells (arrow) and **(c)** a PD patient, showing again very few TLR2 positive cells (arrow), similar to controls, **(a’-c’)** represent higher magnifications of **(a-c)**; bar **(a-c)** = 100 μm; **(a’-c’)** = 50 μm.

**Figure 10:**
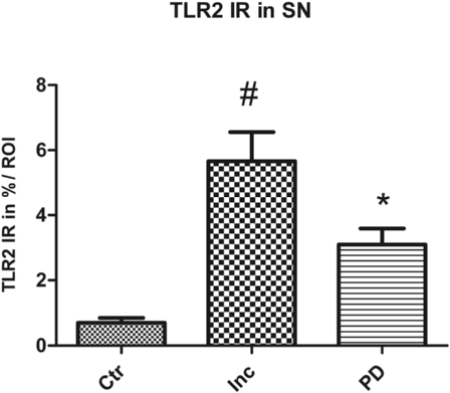
**Quantification of TLR2 immunoreactivity (IR) in the substantia nigra (SN) of control subjects, iLBD cases and PD patients.** A significant increase was found in TLR2 IR in the SN of iLBD cases compared to control subjects and PD patients (#p < 0.05 vs Ctr, PD). In PD patients, TLR2 IR was significantly increased in the SN compared to the SN of control subjects (*p = 0.0001 vs Ctr). Data represent mean ± SEM.

**Figure 11:**
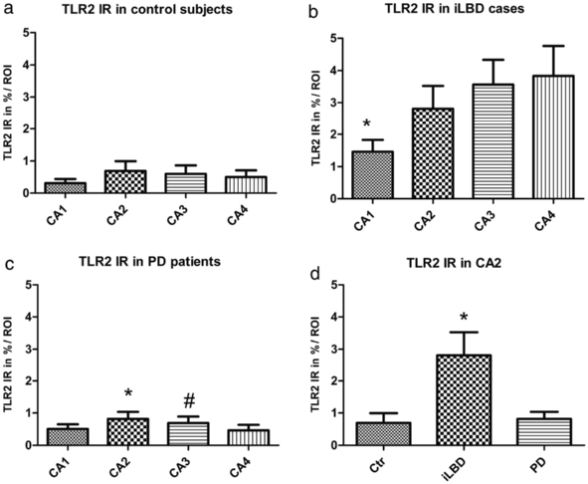
**Quantification of TLR2 immunoreactivity (IR) in the hippocampus (HC) of control subjects, iLBD cases and PD patients. (a)** No significant differences in TLR2 IR were present between the different hippocampal regions within control subjects, **(b)** a significant increase in TLR2 IR was found in CA2, CA3 and CA4 compared to CA1 within iLBD cases (*p < 0.05 vs CA2, CA3, CA4). **(c)** In PD patients TLR2 IR was overall found at lower levels that were significantly increased in CA2 relative to the CA1, CA3 and CA4 (*p < 0.02 vs CA1, CA3, CA4) and also in CA3 compared to CA4 (^#^p < 0.02 vs CA4). **(d)** When TLR2 IR in all four hippocampal subregions was taken together, a significant increase was found in the iLBD cases compared to control subjects and PD patients (data shown for CA2, *p < 0.02). Data represent mean ± SEM.

In control subjects, only very few TLR2 positive cells and processes were observed in the SN and HC (Figure 8a, a’; Figure 9a, a’). In iLBD cases, however, TLR2 IR was prominent and widespread throughout the SN and total HC, and significantly increased relative to control subjects in both regions (Figure 8b, b’; Figure 10; iLBD vs Ctr p = 0.000; Ctr Mean = 0.7 ± 0.2; iLBD Mean = 5.7 ± 0.9; shown for CA2 Figure 9a, b, b’; Figure 11d; iLBD vs Ctr p = 0.018; Ctr Mean = 0.7 ± 0.3; iLBD Mean = 2.8 ± 0.7). This upregulation in the HC was not CA2 specific, but observed in all hippocampal CA regions (Figure 11a-c). However, within iLBD cases, TLR2 IR was significantly higher in the CA2, CA3 and CA4 as compared to CA1 (Figure 11b; CA1 vs CA2/CA4/CA3 p = 0.028; iLBD CA1 Mean = 1.5 ± 0.4; CA2 Mean = 2.8 ± 0.7; CA3 Mean = 3.8 ± 0.9; CA3 Mean = 3.6 ± 0.8).

In PD, TLR2 expression was significantly decreased compared to iLBD cases in both the SN and HC (SN Figure 8a-c, a’-c’; Figure 10; iLBD vs PD p = 0.018; iLBD Mean = 5.7 ± 0.9; PD Mean = 3.1 ± 0.5; HC/CA2 Figure 9a-c, a’-c’; Figure 11d; iLBD vs PD p = 0.013; PD Mean = 0.8 ± 0.2). However, TLR2 IR in the SN of PD patients remained significantly elevated compared to control subjects (Figure 10; Ctr vs PD p = 0.000; Ctr Mean = 0.7 ± 0.2; PD Mean = 3.1 ± 0.5). In contrast, in all pyramidal layers of the HC of PD patients, TLR2 IR was low and comparable to the level of control subjects, and TLR2 IR only in the CA2 was slightly but significantly higher relative to the other CA regions (Figure 11c; CA2 vs CA1/CA4/CA3 p < 0.02; CA2 Mean = 0.8 ± 0.2; CA1 Mean = 0.5 ± 0.2; CA4 Mean = 0.5 ± 0.2; CA3 Mean = 0.7 ± 0.2).

### Colocalization of TLR2 with microglial cells in HC and SN

**Figure 12:**
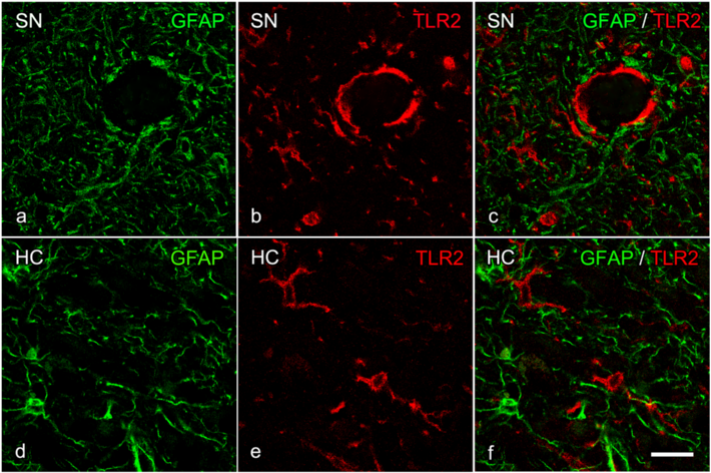
**Absence of colocalization of TLR2- and GFAP immunopositive astrocytes in the substantia nigra (SN) and hippocampal CA2 region. (a-f)** Representative images of confocal laser scanning microscopy failed to reveal any colocalization **(c, f)** between GFAP (**a**, **d**; green) and TLR2 (**b, e**; red) in the **(a-c)** SN and **(d-f)** CA2 of iLBD cases; bar **(a-f)** = 20 μm.

**Figure 13:**
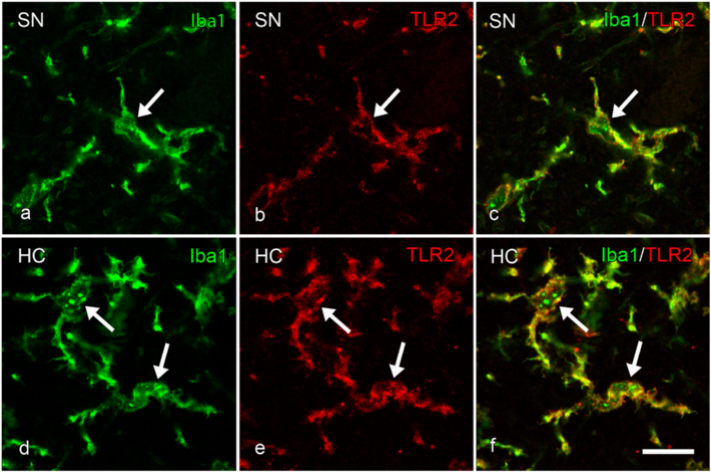
**Colocalization of TLR2- and Iba1 immunopositive primed/reactive microglia in the substantia nigra (SN) and hippocampal CA2 region of iLBD cases. (a-f)** Representative images of confocal laser scanning microscopy revealed colocalization (arrow; **c, f**) of Iba1 positive primed/reactive microglia (**a, d**; green) and TLR2 (**b, e**; red) in the **(a-c)** SN and **(d-f)** CA2 of iLBD cases; bar **(a-f)** = 10 μm.

**Figure 14:**
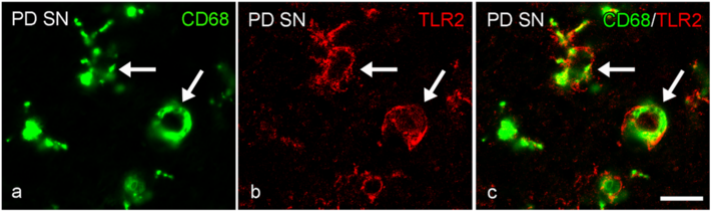
**Colocalization of TLR2- and CD68 immunopositive amoeboid microglia in the substantia nigra (SN) of PD patients. (a-c)** Representative images of confocal laser scanning microscopical images revealed colocalization (arrow; **c**) of CD68 positive amoeboid microglia (**a**; green) and TLR2 (**b**; red) in the SN of PD patients; bar **(a-f)** = 10 μm.

To determine which cells express TLR2, double labeling revealed that TLR2 was not expressed by GFAP positive astrocytes in the SN or HC (Figure 12). However, clear colocalization was found between Iba1 positive primed/reactive microglia and TLR2 IR, in almost all TLR2 positive cells in the HC and SN (Figure 13). Finally, to determine whether amoeboid microglia also express TLR2, double labeling for TLR2 and CD68 revealed that indeed TLR2 IR was present in CD68 positive amoeboid microglia in the SN of PD patients (Figure 14).

## Discussion

**Figure 15:**
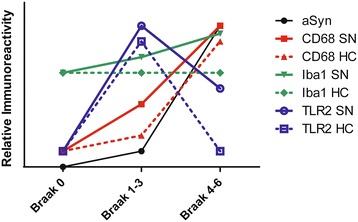
**Schematic summary of the different patterns of α-synuclein pathology and microglial phenotypes and activation (Iba1, CD68, TLR2) in the hippocampal CA2 region and SN during disease progression in PD.**

In the present study, we investigated different microglial phenotypes, and TLR2 expression in relation to the α-synuclein pathology in the SN and HC of iLBD cases, considered a prodromal state of PD, and of established PD patients. Clear differences were present in microglial phenotypes between the SN and HC. The amoeboid type was found to parallel the prominent α-synuclein pathology in the SN and hippocampal CA2 subregion in PD. TLR2 expression was strongly increased in primed/reactive microglia of the iLBD cases. Unexpectedly, in PD patients, TLR2 IR remained upregulated in the SN, but was reduced to control levels in the HC. Thus, TLR2 expression in microglia occurs in a disease stage-dependent manner that differs between the HC and SN. The high levels in iLBD cases and not in PD patients suggest an early activational response to the development of PD pathology. As such, our results confirm, for the first time in human brain, recent in vitro and experimental studies indicating that TLR2 is an important player in the neuroinflammatory responses during PD progression. A general scheme representing the differences in microglial phenotypes and TLR2 expression in the SN and HC during disease progression in PD, as represented by the Braak PD stages, is summarized in Figure 15.

In addition to its well established pattern in the SN, α-synuclein pathology was prominent in the HC, a brain region involved in cognition and affective symptoms that have been implicated in the non-motor symptomatology and frequent dementia and depression in PD patients [5,7]. In line with a previous study, α-synuclein pathology in the HC was largely confined to the CA2 subregion [54]. We observed that this selective vulnerability was independent of whether PD patients had been diagnosed with dementia. Although only few studies have so far implicated CA2 in PD symptomatology, the selective localization of α-synuclein pathology to this subregion and its recent involvement particularly in social forms of memory may hold some promise for a better interpretation of the clinical symptoms [48,55,56].

While general microgliosis has been repeatedly demonstrated in several pathological sites in PD, including the SN, HC and OB, little was known about the brain region-specific phenotypes of microglia and differences between presymptomatic and established PD [23,57,58]. In both the SN and HC of PD patients, the number of activated, CD68 positive, amoeboid microglial cells was increased. As in the HC, this increase was present especially in the CA2 and CA3, notably in close proximity to α-synuclein pathology; this suggests that an inflammatory response occurs to these neuropathological alterations, similar to the SN. Strikingly, increases in amoeboid microglia in the SN were observed in iLBD cases too, in which little, if any, DAergic cell loss was present postmortem, nor had any clinical motor symptoms been apparent during these patients’ lives [51]. This is of importance since it contributes to the debate whether microglial activation is merely a reaction to neuronal cell death or whether these cell respond to other (early) pathological events [13] and therefore actively participate in the pathological processes before cell death occurs, as suggested by our observations in the SN and HC. In the HC however, amoeboid microglia were not increased in iLBD cases, consistent with the fact that the HC at this stage (Braak PD 1–3) is still devoid of any α-synuclein deposits. This is different from the SN that already shows prominent α-synuclein deposits by then. It indicates that α-synuclein deposits may trigger a morphological transition into amoeboid microglia before actual cell loss becomes apparent and is in line with observations in the anterior olfactory nucleus [23], and with experimental studies showing that α-synuclein can trigger microglial activation [20,41,47].

Interestingly, in the HC, the total density of Iba1 positive microglia was not different between the subject groups, whereas in the SN an increase was observed in PD patients compared to control subjects. The phenotype of the Iba1 positive ramified microglia had changed into primed/reactive Iba1 positive microglia in the HC and SN of both the iLBD cases and PD patients, notably irrespective of the presence of α-synuclein pathology. Together, this suggests that Iba1 positive ramified microglia may respond to a stimulus other than accumulated or aggregated α-synuclein, that likely is independent of the local SN or HC environment and is already present during the prodromal stage. Possibly, the primed microglial morphology represents microglia, which are in a more sensitive state to subsequent stimuli, commonly referred to as ‘second hit’ [30,31,59]. Although the exact nature of such a stimulus awaits future studies, oligomeric or soluble forms of α-synuclein are attractive candidates in this respect.

To further characterize the spatio-temporal changes in microglial phenotypes during PD progression, and to identify possible underlying mechanisms, we focused on TLR2, an important member of the TLR family that was recently implicated in microglial activation in PD [41,46]. To rule out any possible differences in microglial activation and TLR2 expression due to AD pathology, NFT and Aβ scores were matched between control subjects, iLBD cases and PD patients. We found TLR2 to be strongly increased in the SN and HC of iLBD cases, but not in PD patients. Recent in vitro and in vivo animal studies have shown that α-synuclein oligomers can activate microglia via TLR2 and thereby stimulate NF_κ_B-mediated pro-inflammatory cytokine production [41,47]. Also, microglial cells treated with α-synuclein significantly enhance their TLR2 expression [35]. Since we found the Iba1 positive primed/reactive phenotype to express TLR2 particularly [29], this suggests that priming of microglial cells, possibly by α-synuclein oligomers, could involve TLR2. The upregulation of TLR2 we found in iLBD cases will then most likely reflect an early activational response of microglia to e.g. α-synuclein oligomers, prior to the development of extensive PD pathology, i.e. when neuronal cell death is still barely present. Furthermore, there only seemed to be a topical relation between α-synuclein accumulation and TLR2 expression in the SN. In the HC, TLR2 expression was widespread and not confined to the CA2 region, suggesting that the factors causing TLR2 upregulation are present in a more widespread distribution in the HC, consistent with a role for e.g. oligomeric forms of α-synuclein. This hypothesis is supported by previous in vitro studies where pre-conditioning microglia with abnormal α-synuclein strongly affected their subsequent TLR2 mediated response [44]. A subsequent challenge (‘second hit’), like actual α-synuclein deposits and/or DAergic cell loss in the SN of PD patients, could then change the primed phenotype into an amoeboid one, as we observed. This is also in line with the increase in amoeboid microglia in CA2 and CA3, where α-synuclein deposits are abundant.

In the HC of PD patients, TLR2 IR was reduced to the level of control subjects, whereas in the SN, its expression remained significantly increased. This difference in expression may reflect the absence of a continuous stimulus, like neuronal loss, that is not apparent in the HC, but does occur in the SN. Indeed, whereas some hippocampal atrophy has been observed by MRI [60,61], neuronal loss is not present in the HC of PD patients [62]. Interestingly, in the CA2, TLR2 expression was still significantly elevated. This also supports the idea that a second stimulus, e.g. α-synuclein deposits, after an early pathological stimulus in iLBD cases, may further trigger TLR2 and microglial activation in PD.

Although the primary role of microglia may be to clear toxic proteins and thereby protect neurons, TLR-2 mediated activated microglia have taken on different or additional roles in chronically diseased brains. So far, most studies interpret microglial activation by TLR2 as a classical microglial activation profile that gives rise to the secretion of mainly pro-inflammatory cytokines [41,63,64]. However, other studies suggest that receptors like TLR2 can be considered as a ‘gateway’ in their functionality that can influence the balance between e.g. phagocytic and pro-inflammatory microglial activity [63]. A high expression level of such receptors would then promote an alternative route of activation (e.g. an anti-inflammatory profile) and phagocytosis, whereas low levels could induce a pro-inflammatory state. It is as yet unknown whether this concept would also apply to TLR2 expression in PD, but it could have important implications for therapy [65]. The final consequences of the TLR2 activation we report here may however, also depend on additional factors, like the assembly state of the protein ligand, the duration of stimulation and the repertoire of co-receptor and adaptor proteins that interact with TLR2 [41].

In summary, we report region-specific differences in the expression of different microglial phenotypes in the SN, a classically affected brain region involved in motor symptoms in PD, and in the HC, a brain region relevant for some of the non-motor symptoms, like dementia, in PD. Besides differential changes in microglial activation and phenotypes in the SN and hippocampal CA2 of PD patients, TLR2 is strongly expressed in primed/reactive microglia in iLBD cases, considered a prodromal state of PD, in both the HC and SN. Understanding how microglia responses and activation differs between regions and how they change during disease progression will improve our understanding of the role of microglia in neurodegeneration and neuroprotection in general, and of their role in PD pathology in particular. Since neuroinflammatory responses are in principle modifiable, such approaches may help to develop new drugable targets or adjunctive therapies for PD-related symptoms.

## Abbreviations

PD: Parkinson’s disease
AD: Alzheimer’s disease
MS: Multiple sclerosis
SN: Substantia nigra
HC: Hippocampus
CA: Cornus ammonis
OB: Olfactory bulb
AON: Anterior olfactory nucleus
IL: Interleukin
iLBD: Incidental Lewy body disease
LBs: Lewy bodies
LN: Lewy neurites
α-synuclein: Alpha-synuclein
Aβ: Amyloid-beta
NFT: Neurofibrillary tangles
TLR: Toll like receptor
TNFα: Tumor necrosis factor-alpha
MPTP: 1-methyl-4-phenyl-1,2,3,6-tetrahydropyridinel
Iba1: Ionized calcium binding adaptor molecule 1
GFAP: Glial fibrillary acidic protein
HPtau: Hyperphosphorylated tau
IR: Immunoreactivity
